# Optical tracking and laser-induced mortality of insects during flight

**DOI:** 10.1038/s41598-020-71824-y

**Published:** 2020-09-09

**Authors:** Matthew D. Keller, Bryan J. Norton, David J. Farrar, Phil Rutschman, Maclen Marvit, Arty Makagon

**Affiliations:** Intellectual Ventures Laboratory, Bellevue, WA USA

**Keywords:** Entomology, Biophotonics, Imaging and sensing, Ecology

## Abstract

Addressing the need for novel insect observation and control tools, the Photonic Fence detects and tracks mosquitoes and other flying insects and can apply lethal doses of laser light to them. Previously, we determined lethal exposure levels for a variety of lasers and pulse conditions on anesthetized *Anopheles stephensi* mosquitoes. In this work, similar studies were performed while the subjects were freely flying within transparent cages two meters from the optical system; a proof-of-principle demonstration of a 30 m system was also performed. From the dose–response curves of mortality data created as a function of various beam diameter, pulse width, and power conditions at visible and near-infrared wavelengths, the visible wavelengths required significantly lower laser exposure than near infrared wavelengths to disable subjects, though near infrared sources remain attractive given their cost and retina safety. The flight behavior of the subjects and the performance of the tracking system were found to have no impact on the mortality outcomes for pulse durations up to 25 ms, which appears to be the ideal duration to minimize required laser power. The results of this study affirm the practicality of using optical approaches to protect people and crops from pestilent flying insects.

## Introduction

Insects continue to have major impacts on both human health and agricultural productivity around the world. The effects of mosquitoes on human health are well documented, as transmission of malaria, Zika, dengue, chikungunya, and other diseases impact millions of people every year^1–6^. Losses in agricultural productivity due to insects such as the Asian Citrus Psyllid (ACP, *Diaphorina citri*)^7^, spotted wing Drosophila (SWD, *Drosophila suzukii*)^8^, and Queensland fruit fly (*Bactrocera tyroni*)^9,10^ are typically less publicized than impacts on human health, but they remain serious economic issues that cannot always be addressed via traditional means, including the use of chemical pesticides.

Among the most promising new technologies to study and monitor insects is the branch of imaging known as computer (or machine) vision, as recently reviewed by Manoukis et al*.*^11^. Several groups have explored combining a computer vision system to identify and track certain insects, typically mosquitoes, with a laser system that can disable or kill the identified insect. Guoqing et al*.* demonstrated a computer vision tracking system and discussed the addition of a lethal laser but did not show this step^12^. Hu et al*.* recently demonstrated a system using two separate lasers to track and shoot down flying mosquitoes at a distance of 20 cm^13^. Although an interesting result, this study used a small sample of targets and laser conditions, and only worked over a very short distance from the optics to the mosquitoes.

As previously described by the authors^14,15^, our concept for an all-optical system designed to identify and disable small flying insects over a wide area is known as the Photonic Fence. In brief, it defines a zone approximately 30 to 100 m long, < 1 m wide, and 3 m tall in which a machine vision system can detect and identify small flying insects based on size (adjusting for the calculated z-position of the insect within the zone), time of day, wing-beat frequency, and/or other factors. Once the system has identified a target insect, a laser sub-system then kills or otherwise disables the target via a short (~ 25 ms) pulse of light, preferably at a “retina-safe” wavelength in the near infrared region. A separate safety system ensures that the laser cannot fire if people or other animals are within a short distance of the area targeted for exposure.

In an earlier study^15^, we focused primarily on determining the optimal laser parameters required to kill or disable *Anopheles stephensi* mosquitoes, which were identified as a good model organism due to their size, role in disease spread, and robustness in lab settings. These studies allowed us to determine several regimes of laser-target interaction^16^, such that we could make generalizations about the effectiveness of various wavelengths, laser spot diameters, pulse energies, and pulse durations. Its primary limitation was that the mosquitoes were anesthetized at the time of laser dosing, which allowed the laser doses to be targeted with excellent accuracy, but is not representative of a realistic use case.

In this study, we expanded the investigation into the more realistic setting of subjects that were freely flying, though still within limited volumes inside transparent cages. Starting from the laser exposure regimes defined as most promising from the anesthetized study, our goal was to determine the sets of laser parameters (wavelength, spot diameter, pulse duration, pulse energy) that offered the best combination of target mortality, system cost, and safety. Concurrently, we sought to determine several key parameters required for accurately tracking and targeting the subjects while they were flying during the laser pulse, as well as the interactions between the laser parameters and tracking characteristics. *A. stephensi* were again used as a model organism for most studies, but other species including ACP, SWD, and the *Culex pipiens* mosquito were explored as well. To our knowledge, this study thus presents the first comprehensive, systematic study to determine the optimal conditions for tracking and disabling multiple flying insect species with an all-optical system, including over relevant real-world ranges.

## Results

### In-flight dosing system

**Figure 1:**
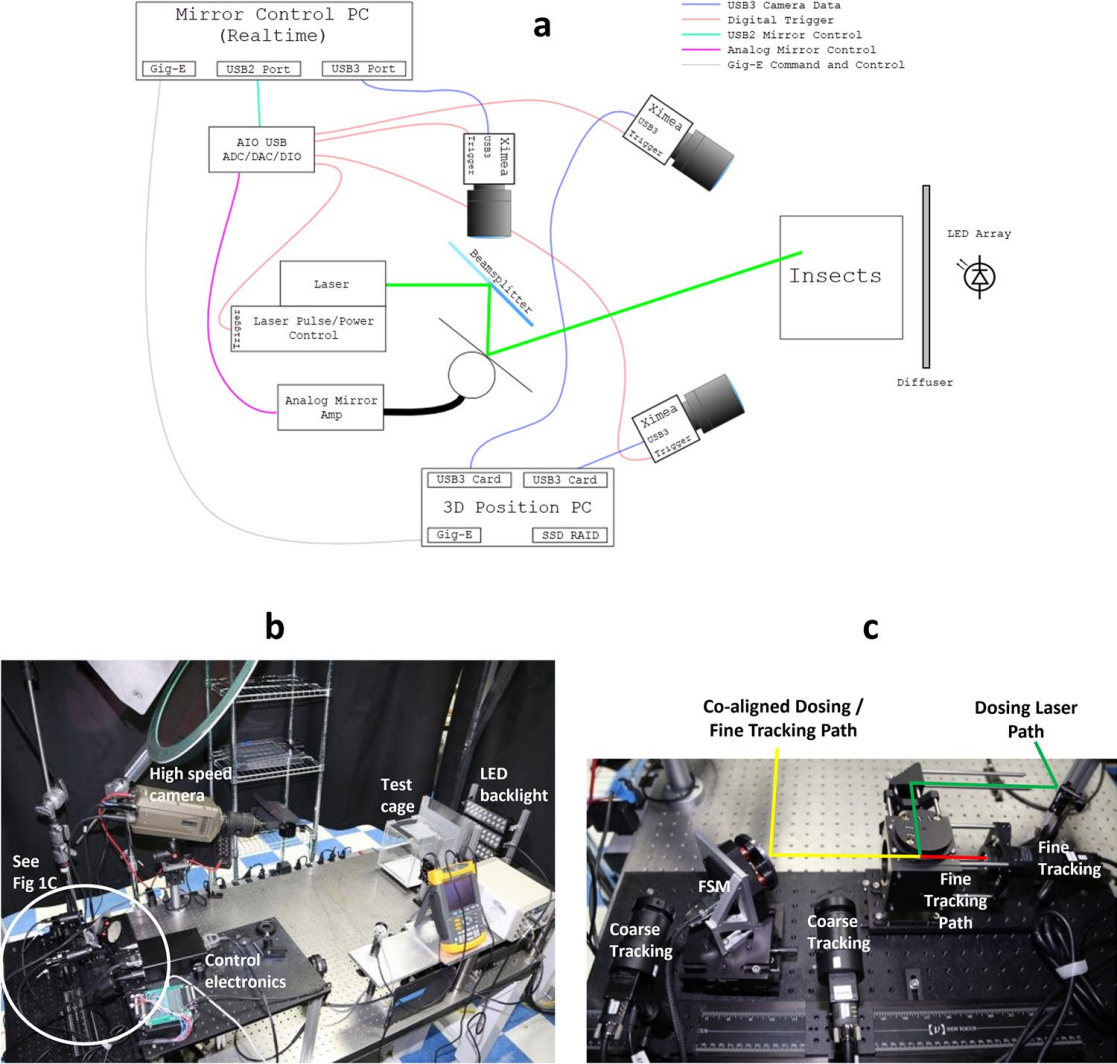
Schematics and images of in-flight dosing setup. (**a**) Schematic (not to scale) of primary dosing system components and communication lines among them. Note that the mirror control and 3D position subsystems were run on separate kernels within the same PC. (**b**) Image of complete system setup including the test cage location, LED backlighting, and control electronics. Cameras for coarse and fine tracking, the fast scanning mirror (FSM), and dosing laser path are contained in the white circle and better seen in (**c**) close-up image of core tracking and dosing system components and alignment among them. Red line represents fine tracking image path, green line the dosing laser path (laser not shown), and yellow line the combined fine tracking and dosing laser path.

The system used in this work is detailed in Fig. 1. It can be broken down into three primary modules: (1) a “coarse tracking” system that uses a pair of stereoscopic cameras to identify the three dimensional location of a target subject, which is passed on to (2) the “fine tracking” system that uses a single higher speed camera and a fast scanning mirror (FSM) to keep the target in the middle of the field of view (FOV) of the camera using a proportional-integral-derivative (PID) control loop, and (3) the laser dosing system that fires the laser pulse, which is co-aligned with the fine tracking system to ensure the laser pulse is accurately applied to the subject even while it is moving. For both the coarse and fine tracking systems, subjects are identified by the size of their silhouettes generated from near infrared LED back-illumination or reflection. As demonstrated in Fig. 1b, the subject cages were 20 cm cubes constructed from clear acrylic, but with the side facing the tracking and dosing systems made of borosilicate glass, placed two meters away from the FSM. A high-speed video camera (Vision Research Phantom) was set up to record a small subset of experiments at 2000 frames per second as well. For all experiments, each cage contained only a single subject to be illuminated, or “dosed,” with the laser, and conditions were set such that a subject could be dosed only when it was at least 2.5 to 5 cm away from any wall of the cage to ensure the subject was flying normally, rather than taking wing or alighting.

**Figure 2:**
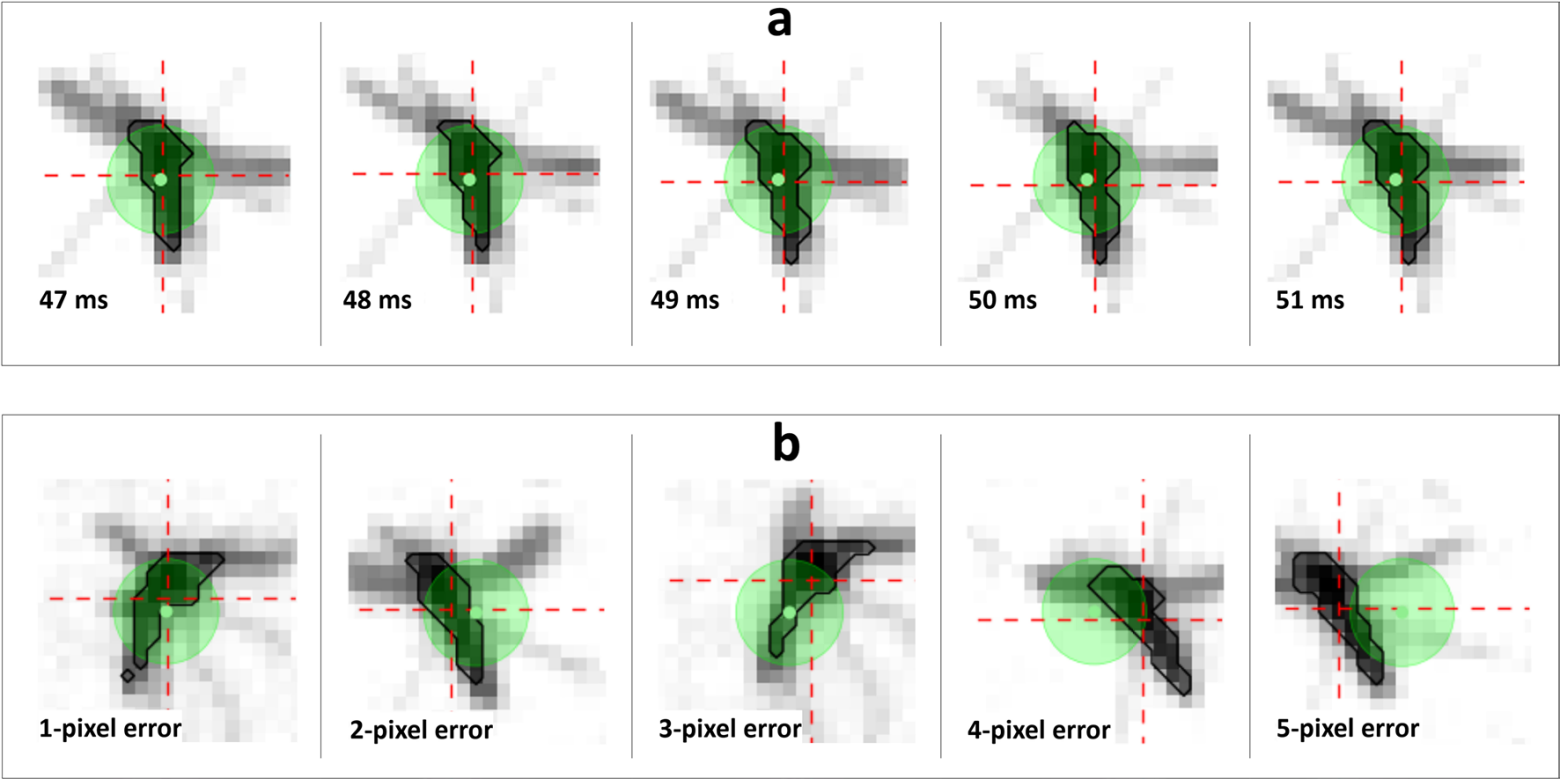
Representative fine tracking camera images of *A. stephensi* silhouettes. In each frame, the approximate outline of the thorax and abdomen is drawn (thick black lines) according to a set pixel intensity threshold. The centroid of this region is then calculated (intersection of red crosshairs) and compared with the center of the camera’s field of view (green dot) to determine the current tracking error and provide input to the fine tracking PID loop controlling the direction of the scanning mirror. The green circle around the green dot represents the spot size of the laser (2.5 mm diameter for all images shown here); occlusion factor represents how much of this laser spot (assuming a Gaussian profile) overlaps with the body of the subject. The images in (**a**) demonstrate a typical time course for a single subject in 1 ms intervals, and those in (**b**) show representative depictions of tracking errors ranging from 1 to 5 pixels for various subjects.

Prior to commencing the laser dosing experiments, a number of parameters were defined to analyze the results, as detailed in Table 1 and Fig. 2. The key outcome, in line with our previous study^15^ and with WHO guidelines on insecticide treatments^17^, was whether the subjects were alive or disabled (i.e. dead or moribund) 24 h after the treatment. To characterize how well the subjects were tracked during the laser pulse, the tracking error was defined as how far the insect’s centroid was from the center of the fine tracking camera’s FOV. Other parameters defined in Table 1 relate to the “occlusion factor,” or how much of the laser beam’s energy (assuming a Gaussian profile) overlapped with the target’s outline, as demonstrated in Fig. 2. From the coarse tracking system’s output of xyz position of the target, we could also define velocity, speed, and both linear and angular acceleration of the subjects before, during, and after the laser pulse.

**Table 1:** Definitions of all parameters tracked or calculated for each in-flight dosing event.

Parameter	Definition	Data source/method
xyz position	Coordinates of subject within test cage	Calculated by coarse tracking system
Flight velocity	Velocity vectors in x, y, z directions	Calculated from Kalman filtered xyz position data
Flight acceleration magnitude	Magnitude of acceleration vector	Derivative of velocity data
Flight speed	Linear speed of flight	Magnitude of velocity vector
Tracking error	Distance in pixels (1 px ~ 250 um) between calculated subject centroid and center of fine tracking camera’s field of view for each frame	Calculated from fine tracking system images
Occlusion factor	Percentage of laser dosing beam that was incident upon target, assuming Gaussian laser spot profile	Laser spot location from center of fine tracking field of view, compared against outline of subject shape from same fine tracking images

### Initial results with 532 nm laser

**Figure 3:**
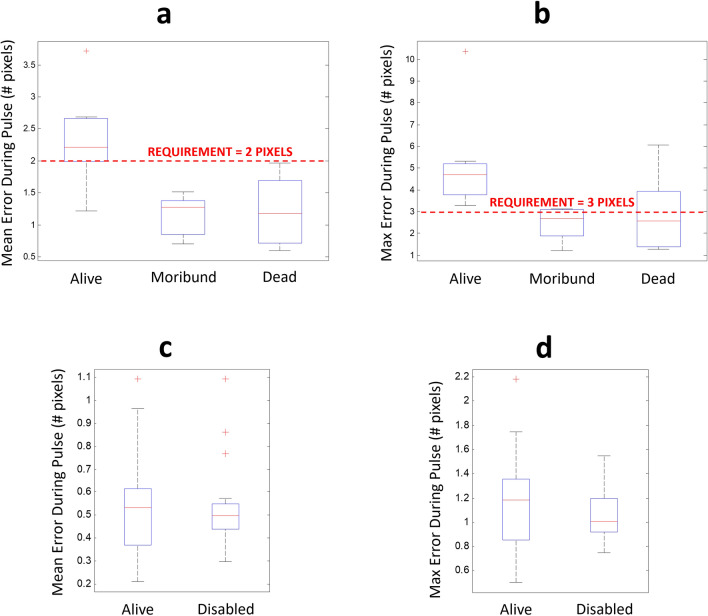
Influence of mean and maximum tracking errors on mortality outcomes. Initial mortality outcomes using LD90 conditions with a 532 nm laser from previous anesthetized work showed strong correlation with (**a**) mean and (**b**) maximum tracking errors over the 25 ms pulse duration. After setting and achieving new tracking error targets of less than 2 pixels mean and 3 pixels max (see Fig. 2b for depictions of overlap between the laser spot and the subject for various error magnitudes), identical experiments no longer showed any correlation with (**c**) mean or (**d**) maximum tracking errors. Moribund and dead outcomes were grouped in (**c**) and (**d**), labeled “Disabled,” and subsequent experiments since they both represent functional kills and are grouped as such in WHO guidelines for insecticide trials.

Initial experiments were conducted with *A. stephensi* subjects and a 532 nm laser (Verdi, Coherent) using parameters for power (3 W), pulse duration (25 ms), and laser spot diameter (2.5 mm) that were defined in our previous work^15^ as an optimal combination of potential cost and mortality performance. As seen in Supplemental Video 1, the system could be quite effective in disabling a flying mosquito with these parameters. Of note from the video, along with flight tracking data for numerous trials (not shown), the 25 ms pulse duration appeared to be short enough that the mosquitoes did not perceptibly alter their flight pattern during the pulse to throw off the tracking algorithm. From Fig. 3a,b, though, the initial system did not have consistent enough tracking performance, such that survival of the subjects was almost entirely dictated by how well the fine tracking system kept the target near the center of its FOV. From this initial experiment on a sample of 80 subjects, we set limits for mean and maximum tracking error during the laser pulse as 2 and 3 pixels, respectively, where each pixel represents ~ 250 μm. Figure 3c,d demonstrates that the system was able to achieve and maintain this performance after modifying the PID loop parameters, and that there was no longer any association between tracking performance and mortality at these conditions. These criteria were evaluated and assured for all mortality data reported in this manuscript (i.e. a Kruskal–Wallis test indicated no significant differences in tracking errors among subjects that survived or were disabled by the laser pulses). Further, Supplemental Fig. S1 shows that flight behavior, in particular speed and linear acceleration, did correlate with tracking accuracy, but that the system was able to meet the noted tracking requirements even at the extremes of flight behavior that could be observed in this study given the restricted flight volumes (though the typical values seen here largely align with available data for other *Anopheles* mosquitoes^18,19^).

**Figure 4:**
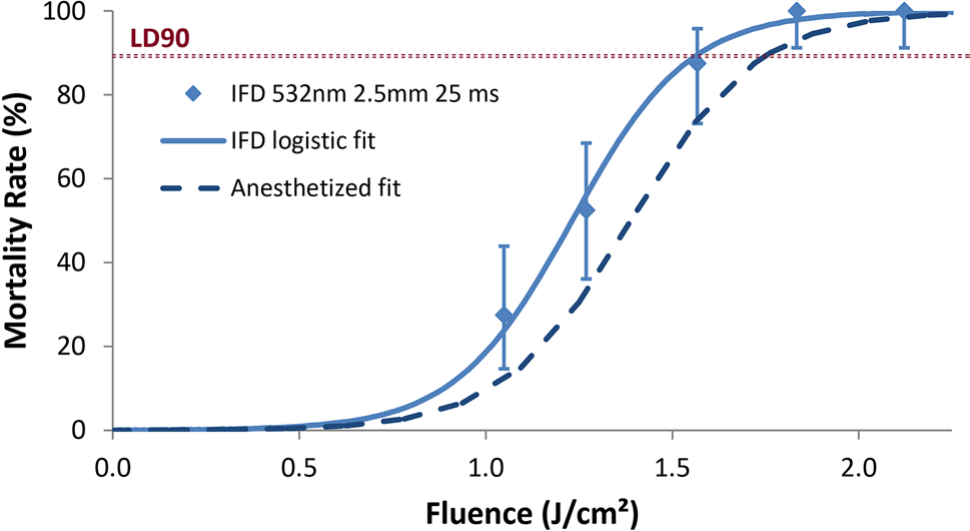
Mortality data and logistic fit (dose–response curve) for IFD with 532 nm, 25 ms pulse duration, and 2.5 mm spot size. Each data point represents the mortality outcome from a set of 40 subjects, and error bars represent 95% confidence intervals from the binomial distribution. The intersections of the dashed horizontal line at 90% mortality with the dose–response curves indicate the LD90 points, which are also presented in Table 2. IFD logistic fit (solid line) had pseudo R^2^ value of 0.99 as shown in Table 2. Dashed curve represents the fitted dose–response curve for the same laser conditions from previous anesthetized dosing work for comparison.

Mortality results were analyzed as in Fig. 4. Each data point represents the mortality outcomes (i.e. percentage of targets that were dead or moribund at 24 h, assuming acceptable control mortality < 5%) of 40 subjects, dosed one at a time with one subject per cage, at a given fluence (laser pulse energy per unit area). Logistic regression curves were then fit to the data to determine LD90 (lethal dose 90, or dose where 90% mortality is expected) for a given set of laser conditions. Figure 4 shows the resulting dose–response curve for the parameters described above—532 nm, 3 W, 25 ms pulse duration, and 2.5 mm spot diameter. For comparison, the dose–response curve using these same parameters for anesthetized mosquitoes in our previous work^15^ is shown as well. Although confidence intervals for the anesthetized fit are not shown for the sake of visual clarity, it is apparent that there were no substantial differences in the dose–response curves for the anesthetized and the in-flight dosing experiments, which utilized the same stock of *A. stephensi* from the same source, and otherwise cared for identically.

### Effects of larger spot size and shorter pulse duration

**Figure 5:**
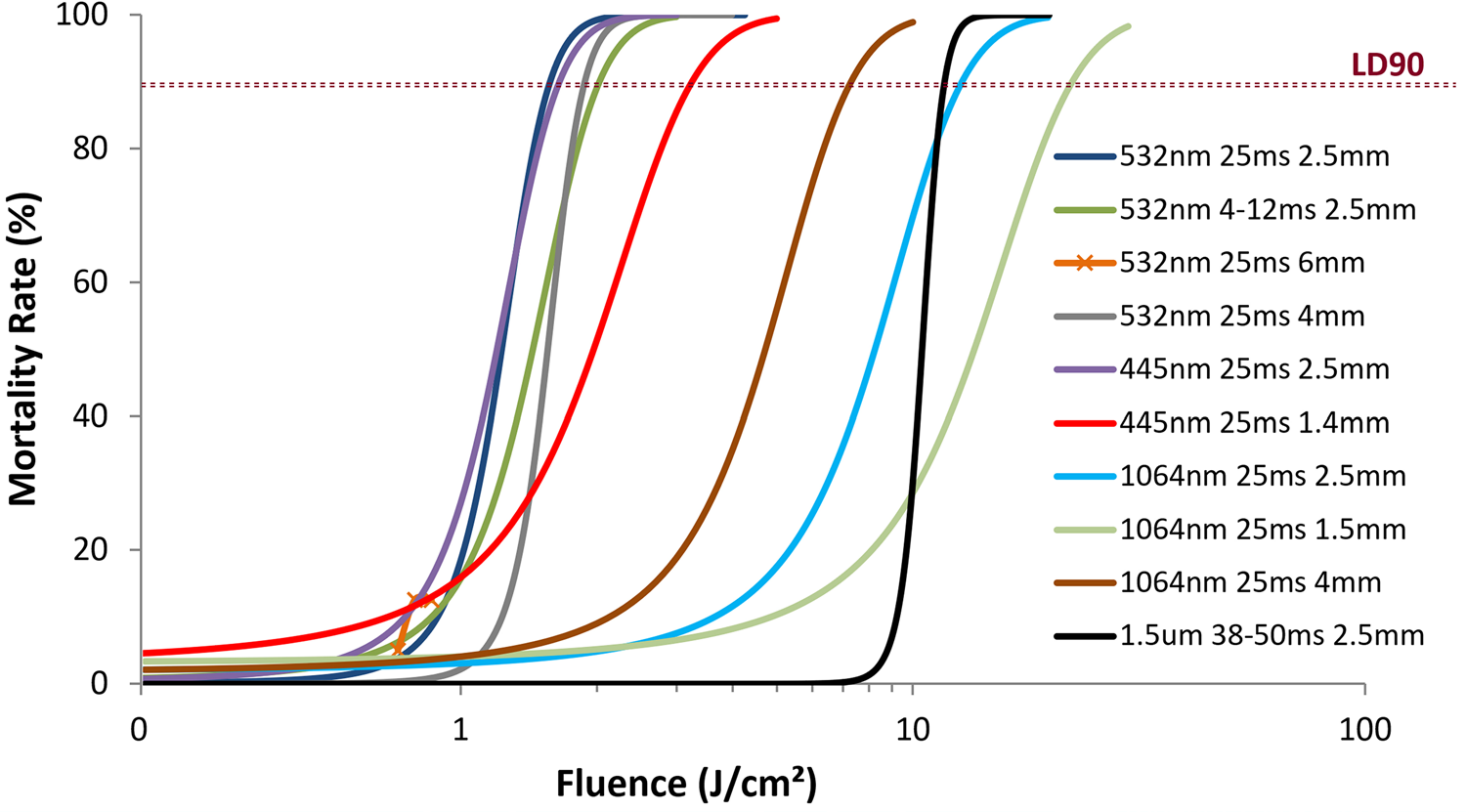
Dose–response curves for all experiments from Table 2, other than the two constant pulse energy tests. The x-axis is plotted on a log scale to allow clear visualization of the curves at both low and high fluence values. Individual data points and error bars not shown for the sake of clarity. The intersections of the dashed horizontal line at 90% mortality with the dose–response curves indicate the LD90 points, which are also presented in Table 2, along with pseudo R^2^ values for the logistic regression fits.

As shown in Table 2 and the left-hand side of Fig. 5, additional experiments explored the impacts of varying other parameters with the same 532 nm laser, in particular examining whether shorter pulse durations or larger spot sizes would result in net reductions of the LD90 fluence. Combinations of both using a higher power with shorter pulse durations, or a higher power but larger spot diameter (to 4 mm) led to slight increases in the LD90. Of note, the 6 mm spot diameter offered limited data points due to the maximum power available from the laser. Figure 5 also shows two curves using a 3.5 W blue diode laser (445 nm, Nichia NDB7K75), which would be a more cost-effective implementation source compared with the lab-grade 532 nm Coherent laser. The two sources were expected to provide similar laser-mosquito interaction regimes, and indeed, Fig. 5 shows that the dose–response curves for both sources are nearly identical when implemented with 3 W, 2.5 mm spot diameters, and 25 ms pulse durations. Because of the limited blue diode power, the spot diameter was not increased, but one experiment was completed with a 1.4 mm spot diameter and 25 ms pulse duration; this dose–response curve looked markedly different from the other visible wavelength curves in Fig. 5, with an LD90 > 2 × that of the other experiments at 445 or 532 nm. Note that this greater LD90 fluence value for the smaller spot still represented a lower pulse energy than required for the larger spots given the spot size differential.

**Table 2:** List of in-flight dosing experimental conditions and resulting LD90 fluence values for *A. stephensi* subjects.

Wavelength of laser (nm)	Varied parameter	Set parameters	LD90 fluence (J/cm^2^)	Pseudo R^2^ of logistic fit
532	Power	Pulse duration = 25 ms Beam diameter = 2.5 mm	1.6	0.99
532	Pulse duration	Power = 8.5 W Beam diameter = 2.5 mm	2.1	0.97
532	Power	Pulse duration = 25 ms Beam diameter = 6.0 mm	Could not be determined	N/A
532	Power	Pulse duration = 25 ms Beam diameter = 4.0 mm	1.9	0.94
532	Pulse duration / power (to keep pulse energy constant)	Beam diameter = 2.5 or 4 mm Pulse energy = LD75 from above	N/A	N/A
445	Power	Pulse duration = 25 ms Beam diameter = 2.5 mm	1.6	0.92
445	Power	Pulse duration = 25 ms Beam diameter = 1.4 mm	3.2	0.93
1,064	Power	Pulse duration = 25 ms Beam diameter = 2.5 mm	12.9	0.95
1,064	Power	Pulse duration = 25 ms Beam diameter = 1.5 mm	7.3	0.88
1,064	Power	Pulse duration = 25 ms Beam diameter = 4.0 mm	22.5	0.93
1,064	Pulse duration / power (to keep pulse energy constant)	Beam diameter = 2.5 mm; Pulse energy = LD75 from above	N/A	N/A
1,570	Pulse duration	Power = 11 W Beam diameter = 2.5 mm	11.7	0.98

Unless otherwise noted, the varied parameter also had the effect of varying pulse energy, and therefore fluence as the independent variable. Pseudo R^2^ values assess the quality of the logistic regression fit used to determine the LD90 fluence values.

### Dosing with near infrared wavelengths

Two near infrared (NIR) laser sources were examined as well—a custom constructed 1,064 nm (1 μm) fiber laser with a maximum output of 30 W and a commercial 1,570 nm (1.5 μm) fiber laser (IPG Photonics) with a maximum deliverable output of 11 W. The right-hand side of Fig. 5 shows the dose–response curves for these laser sources in addition to those using visible wavelengths. All of the infrared experiments resulted in significantly higher LD90 fluence levels compared with the visible lasers, and given the log scale of Fig. 5, varying experimental conditions with the 1 μm source led to comparatively larger changes in LD90 compared with the visible sources. As with the blue diode, the small spot (1.5 mm) had the highest LD90 fluence, but unlike the visible lasers, the larger spot (4 mm) offered a lower LD90 fluence compared with the baseline 2.5 mm. For the 1.5 μm source, there was insufficient mortality at the maximum power available from this source with a 2.5 mm spot and 25 ms pulse duration, so Fig. 5 shows a curve using slightly longer pulse durations to make up the additional fluence required. From the available data, it appears that the LD90 fluence levels for the 1 μm and 1.5 μm sources are at least at reasonably comparable levels.

### Effects of longer pulse durations with lower power

**Figure 6:**
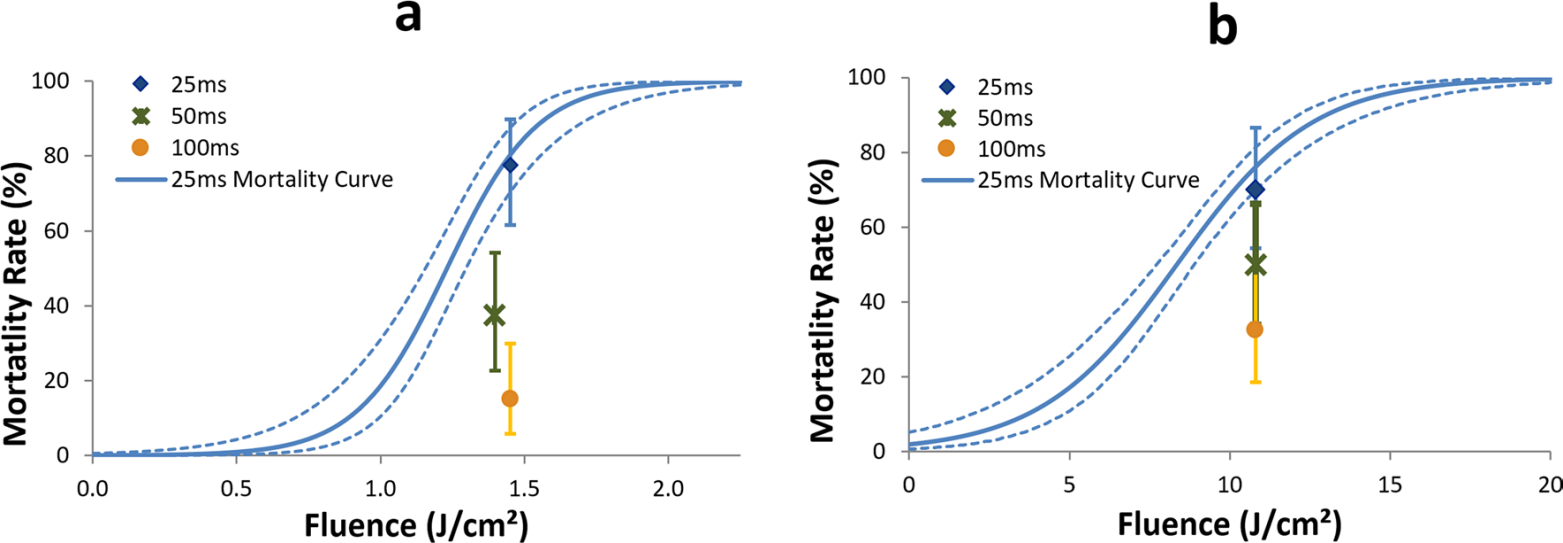
Mortality results for experiments with constant pulse energy achieved by proportionally adjusting the power according to pulse duration (25, 50, and 100 ms). Dosing was performed with both the (**a**) 532 nm and (**b**) 1,064 nm lasers, both using a 2.5 mm spot size, and set to the approximate LD75 fluence value from the dose–response curves (solid curves with dashed line 95% confidence intervals of logistic regression fit) from the corresponding 25 ms experiments. Error bars on individual points represent 95% confidence intervals of exact binomial probabilities. Statistical differences among mortality at the various pulse durations summarized in Table 3. The slight offsets on the x axis in (**a**) stem from slight changes in the beam spot size as a function of power.

We then further explored the effects of increasing the pulse duration to determine whether this could relax the optical power required from the laser source, given that laser cost is correlated with output power. Figure 6a,b show the results for the 532 nm and 1,064 nm sources, respectively, with spot diameters of 2.5 mm and pulse duration set to 25, 50, or 100 ms. In all cases, the optical power was adjusted according to the pulse duration to supply a constant pulse energy, and therefore fluence at approximately the LD75 level determined from previous experiments, which was chosen to ensure a low likelihood of any data point being 100% mortality (i.e., a saturated signal). As evidenced from Fig. 6a and Table 3, at 532 nm there was a significant drop off in mortality at the longer pulse durations, although the effect is smaller going from 50 to 100 ms compared with the initial drop from 25 to 50 ms. Similar, but smaller magnitude effects were seen with the 1 μm source in Fig. 6b and Table 3. Supplemental Fig. S2 shows that the tracking accuracy for these experiments somewhat mirrors the mortality performance, with a notable decrement from 25 to 50 ms but no significant change from 50 to 100 ms.

**Table 3:** Results of χ^2^ tests for mortality equivalence among experiments with constant pulse energy but varied amounts of power and pulse duration (25, 50, and 100 ms).

Comparison	*P* value for 532 nm	*P* value for 1,064 nm
All pulse durations	< 10^−5^	< 6 × 10^−5^
25 vs. 50 ms	0.0003	0.07
50 vs. 100 ms	0.02	0.1
25 vs. 100 ms	< 10^−29^	0.0008

Dosing was performed with both the 532 nm and 1,064 nm lasers, both using a 2.5 mm spot size, and set to the approximate LD75 fluence value from the experiments described in Table 2. Results correspond to data shown in Fig. 6 as well.

### Comparing dosing performance across other insects

Using the same system and procedures, experiments were also run on SWD and ACP subjects as a way of cursorily exploring laser-insect interaction for species of interest besides *A. stephensi.* For SWD, a full dose–response curve was created for the 1 μm laser with typical exposure settings (2.5 mm spot diameter and 25 ms pulse duration). The LD90 fluence from this work was 8.6 J/cm^2^, compared with 12.9 J/cm^2^ for *A. stephensi* under the same conditions. Tracking performance on SWD subjects was comparable to that for *A. stephensi*, given that flight parameters such as speed and acceleration were comparable as well. With ACP subjects, inducing them to fly in the cages was very difficult. As such, we could not process a sufficient number of subjects to create a proper dose–response curve. Based on the limited data available, and as demonstrated in Supplemental Video 2, the system as set for the mosquito LD90 condition with the 1 μm laser, 2.5 mm spot diameter and 25 ms pulse duration was effective in tracking and disabling the ACP subjects, despite their very different flying behavior (greater speeds and accelerations, limited durations) relative to the other test species.

### Longer range dosing system demonstration

As a final proof of principle test, a long-range version of the system from Fig. 1 was configured. This system worked at a distance of 30 m instead of 2 m, facilitated primarily by modifying the optics used for the coarse and fine tracking systems. Given the reduced flexibility of this system, it was only used with the 1 μm laser with a 2.5 mm spot diameter and 25 ms pulse duration. Rather than acquiring new dose–response curves, which was impractical given the setup’s location in a building ~ 30 km away from the insect-housing chamber, we verified the efficacy of the system using the LD90 conditions established by short-range dosing. Supplemental Video 3 shows this system dosing an *A. stephensi* subject from the same stock as used for short-range testing, and Supplemental Video 4 shows the same for a wild-sourced *Culex pipiens* mosquito obtained in a local pond. Note that the wild *C. pipiens* was substantially larger in size than the lab-reared *A. stephensi*. In all of the limited number of tests of this system for both species (10 *A. stephensi* and 5 *C. pipiens*), the mortality rate was 100%.

## Discussion

This work demonstrated the feasibility of optically tracking freely flying insects and disabling them with a short pulse of laser light. As such, it was an important next step following our work with anesthetized subjects. The mortality results for this “in-flight dosing” relative to the anesthetized work differed by wavelength. Under otherwise identical laser conditions, for the visible wavelengths, LD90 fluence values were comparable between anesthetized and flying subjects, while for infrared wavelengths, the LD90 values were greater for flying subjects relative to anesthetized. The reasons behind this difference are not clear, but could stem from the different regimes of laser-target interaction (i.e., thermal confinement vs. no confinement, which as discussed previously^15,16^ refers to how efficiently a laser pulse heats up the target material during the pulse) expected from the two wavelength ranges, and how these interaction regimes may lead to damage that kills or otherwise disables the target subjects.

Since laser cost is highly correlated with optical power for a given wavelength and laser technology, it is important to use as little power as possible for the dosing subsystem while also optimizing the spot size. From our previous work, and confirmed again for in-flight dosing in Fig. 5, there is no apparent value in using pulses shorter than 25 ms, as the LD90 fluence does not markedly change, and thus the optical power requirement increases for shorter pulses. In addition, flight behavior was not observed to change substantially during a 25 ms pulse, which makes it more straightforward to continue tracking the subject during the pulse. As demonstrated in Fig. 6 and Table 3, reducing the power and using a longer pulse (to match the pulse energy of the 25 ms pulse) does not seem like a promising approach either, as mortality was significantly reduced under these conditions for both visible and infrared light. The mortality dropped off more dramatically for visible light than for infrared light, presumably because under these conditions, the visible light was near the boundary between thermal and no confinement zones, while the infrared light was always in the no confinement regime. That is, the efficiency of depositing energy into the target during the pulse dropped more substantially as a function of pulse duration for visible light than for infrared light. This reduced mortality for longer pulses may also be related to the diminished tracking performance seen during these longer pulses, as demonstrated in Supplemental Fig. S2.

Spot size is also a major consideration for minimizing the power required for the dosing beam. Although a larger beam requires more power to achieve the same fluence, there was a hypothesis that larger beams could still result in a net power savings if they obviated the concern of keeping the laser in a consistent location on the target during the pulse (i.e. the green circle representing the laser spot in Fig. 2 would overlap with the target more consistently), or if some part of the subject irradiated by the larger spot but not the smaller ones were more sensitive to laser-induced damage. From Fig. 5 and Table 2, though, it is apparent that beams larger than 2.5 mm diameter result in a net increase in required power. For example, in Fig. 5 the LD90 fluence for green light showed a modest increase going from a 2.5 mm beam to a 4 mm beam. Even if these curves were statistically equivalent, the 4 mm beam would still require ~ 2.5 times more power or pulse energy than the 2.5 mm beam to achieve the same performance. Elsewhere in Fig. 5, the LD90 for the 1 μm laser decreased from 12.9 J/cm^2^ for the 2.5 mm beam to 7.3 J/cm^2^ for the 4 mm beam, but this still results in a net increase in pulse energy, and therefore power for the larger beam. In contrast, moving from a 2.5 mm beam to a ~ 1.5 mm beam for either blue light or 1 μm light (Fig. 5) led to increased LD90 fluence values, but in ratios that would allow a small net savings in power for the smaller spots. For example, the 1.4 mm spot size for blue light would require ~ 50 mJ of pulse energy, while the 2.5 mm spot size would require ~ 78 mJ pulse energy. This finding must be balanced against the ability to create such small spots in a longer range system, as smaller spots at longer distances would require larger optics and higher beam quality lasers, both of which would add cost to the system.

Ideally, the Photonic Fence system would be applicable to a wide variety of target insects. The current system was developed primarily for anopheline mosquitoes, but it appears to be fairly straightforward to use it on other potential subjects. Without changing any parameters in the tracking system that were optimized for the *A. stephensi* subjects, other than threshold size of target silhouettes, the dosing performance showed excellent results for the limited trials with both SWD and ACP. With SWD, we saw similar tracking characteristics and a decreased LD90 fluence, given their slightly smaller size relative to *A. stephensi*. Despite limited success in getting ACP subjects to fly, the system was very effective on subjects that did take flight even though their flight behavior tended toward faster and greater acceleration characteristics. For other potential real-world targets, it is possible that certain tracking algorithm parameters (e.g. detection threshold) or dosing laser conditions (e.g. power, spot size) would need to be altered to optimize performance.

Since much of this work was performed with an apparatus having a 2 m working distance, it was important to perform a proof of principle demonstration with a longer range (30 m was selected) system as well, since such scales are much more realistic for actual field deployment. Although we were not able to obtain the complete suite of data as for the shorter range experiments, Supplemental Videos 3 and 4 demonstrated that the same approach, with appropriately modified optics, was equally effective on both *A. stephensi* and the somewhat larger, wild-sourced *C. pipiens* mosquito.

With the feasibility of this approach now demonstrated, future work includes value engineering the components required for a longer range system, which includes leveraging a maturing market for near infrared fiber laser sources, particularly at “retina safe” wavelengths around 1.5 μm. These are an attractive choice for the dosing laser given their compact, reliable, and robust nature in addition to their retina safety. Further dosing experiments need to be performed with the longer range system in large enclosures or outdoor settings, where the subjects are able to fly in a completely natural environment; to that end, we have begun work toward a system where the active zone is 30 m long by 0.3 m wide by 3 m tall, all enclosed in a screen house with dimensions of 40 m long by 4 m wide by 3.3 m tall. We also look to perform mixed species tests, where multiple species are in the active zone at the same time, to determine which factors besides size (e.g. morphology, wingbeat frequency, time of day, environmental conditions like wind speed, rain, etc.) may be required to distinguish target subjects from beneficial species. We also continue to refine the concepts behind the safety system that would disable the dosing laser should people or non-target animals come within the system’s hazard zone. In parallel, we will explore how the tracking systems can be modified slightly such that they could be implemented as a stand-alone option for use in entomological research or monitoring applications.

## Methods

### Insects

The primary mosquitoes used in this study were *Anopheles stephensi* clone STE 2, originally from the Malaria Research and Reference Reagent Resource Center (MR4), and provided locally by the Seattle Children’s Research Institute (formerly Center for Infectious Disease Research). Females were separated from males prior to transfer to the IV Lab via local courier. In the Intellectual Ventures Laboratory (IV Lab) insectary, which was maintained at 27°C and 75% relative humidity (for all species), the adult females were fed a solution of 10% dextrose in water via a soaked cotton ball, both before and after dosing experiments.

The spotted wing *Drosophila* (SWD), or *D. suzukii*, were provided by Dr. Elizabeth Beers from Washington State University. The insects were shipped in vials containing a complete food source (Carolina Formula 4–24), and they remained in the vials until experiments began. After dosing, the subjects were provided with the same food source in the dosing cages.

Asian citrus psyllids (ACP) were provided by Dr. Joseph Patt from the United States Department of Agriculture (USDA) Agricultural Research Service. The ACP were shipped along with a small orange jasmine tree (*Murraya paniculata*), which provided both food and habitat for them. After dosing experiments, they were fed with a cotton ball soaked in 30% sucrose and dyed with McCormick green food coloring to make it more visually apparent to the subjects.

The small sample of *Culex pipiens* mosquitoes were obtained by collecting wild larvae from a local pond. After emerging, these mosquitoes were cared for identically to the *Anopheles* mosquitoes.

### Insect handling

At the beginning of each week, ~ 100 *A. stephensi* at a time were mouth aspirated from the transfer containers into each of six 30 cm cube cages, which contained a port for gas tubing. The same day, these aspirated mosquitoes were anesthetized with carbon dioxide via the port for 10–15 min and then gently flicked with an artist’s brush one at a time into the IFD test boxes for use on the following two days. Each IFD box was a 20 cm acrylic cube but with one face consisting of borosilicate glass, and a hinged lid with a mesh-covered cutout to allow airflow and placement of a 10% dextrose-soaked cotton ball for feeding. Each box contained a single subject for dosing, although for each set of 40 test boxes, a “mass control” box was prepared with at least 10 subjects as well that was handled in the same manner as the test boxes. Experiments showed this mass control method provided mortality outcomes equivalent to placing single subjects in individual control boxes, so by using four mass control boxes instead of 40 individual controls, experimental throughput was greatly increased. Dosing experiments were conducted four to five days after shipment to IV Lab, such that our subjects were seven to ten days old (post-emergence) at the time of the dosing experiments.

Other species followed the same general procedure, with minor modifications as required given how the subjects were transported. For example, the SWD tubes were opened inside of the 30 cm cages instead of being aspirated, and the ACP were anesthetized while still on the tree. In all cases, subjects were checked to ensure they were behaving normally prior to being placed in the dosing setup.

### In-flight dosing setup

The IFD system comprises three subsystems – coarse tracking, fine tracking, and laser dosing.

The coarse tracking system consisted of two Ximea cameras (MQ013MG-ON) with attached varifocal lenses (Tamron 12VM1040ASIR), arranged stereoscopically and taking 512 × 512 pixel images at 550 frames per second. The images were analyzed by a dedicated kernel for the 3D location of dark mosquito silhouettes against an LED back-lit ~ 850 nm background (superbrightleds LBIR-850-35). The cameras had 800–880 nm bandpass filters (Omega Optical 840AF80) attached to the front of the lenses to block out any visible light from the room and scatter from the dosing lasers. When the current position of the subject met given criteria (generally greater than 2.5 cm away from any wall), the position was then fed to the separate Realtime kernel on the same PC running the fine tracking subsystem.

The fine tracking system contained one set of camera, lens, and bandpass filter identical to the coarse tracking system. It acquired 64 × 64 pixel images at 1,000 frames per second to provide faster, higher resolution tracking of the subject silhouettes after the coarse tracking identified them as being within the dosing zone. These images were analyzed by the Realtime kernel, which through an Acces I/O USB module (USB-AO16-4A^)^ then controlled the position of a fast scanning mirror (FSM, Optics in Motion OIM2) that aimed to keep the centroid of the subject silhouette in the middle of the camera field of view. This was accomplished via a PID loop control that was optimized to meet the tracking performance during dosing noted above. When the fine tracking system indicated sufficient tracking performance (less than 2 pixel tracking error for a minimum of 50 ms), it checked that the 3D position still met its requirement and then triggered the dosing laser to fire via the Acces I/O module. Fine tracking then continued for up to 300 ms, and coarse tracking continued for up to 500 ms following the completion of the laser pulse.

In the dosing system, the given dosing laser was axially co-aligned with the fine tracking camera via a dichroic beam splitter (Thorlabs DMLP650L for green and blue dosing wavelengths, Thorlabs DMSP100L for 1 μm, DMSP1180 for 1.5 μm) before the combined light path hit the FSM. In this manner, the dosing laser always hit a known portion in the middle of the field of view for the fine tracking camera. Any optical elements to control beam size and shape were placed before the dichroic.

### System calibration

At the beginning of each day of dosing, the coarse and fine tracking systems were calibrated by imaging a set of QR codes placed at positions corresponding to the front and back faces of the IFD cages. This allowed the tracking systems to identify common real-world coordinates from their local coordinates. To make sure the dosing laser was aligned with the fine tracking camera (i.e. proper boresighting), a test box was outfitted with laser viewer cards (Thorlabs VRC4)—one in the middle of the front face and another at the top left corner of the back face of the cage. Sufficient light emitted from the card passed through the dichroic filter and the bandpass filter to be imaged by the fine tracking camera. The alignment routine then compared the center of the emitted light to the center of the camera view while the laser position was adjusted to minimize the difference between the two centers. The FSM rapidly switched the laser between the two locations to simultaneously adjust their alignments; if alignment was good at both locations, the laser and camera were therefore aligned over the entire dosing volume.

### Dosing procedure

One IFD cage at a time was placed in a defined location in view of the IFD apparatus. A virtual active region was created within each box, which extended no closer to any wall than 2.5 cm to ensure the subjects were flying in a normal manner during dosing, and also to make sure that all of the cameras maintained clear views of the subject at all times. To ensure the subjects stayed in the active region during the dosing operation, a trigger zone was defined that began another 2.5 cm inward from the active region boundaries. When the subject flew into the trigger zone, as detected by the coarse tracking subsystem, the system initiated the dosing process to be carried out by the fine tracking and dosing subsystems. If the subject did not enter the trigger zone of its own accord after ~ 1–2 min of the system being armed, it was prodded either with gentle blowing through a port, making sure not to blow so hard that its flight was affected, or with gentle tapping that did not move the box. If the subject continued not to fly for another ~ 1–2 min, it was either skipped for the time being or replaced with a spare subject in a distinct cage.

After a successful triggering/dosing event, the current box was swapped out for a new, undosed one until all subjects for a given data point had been presented. Mass control boxes were placed in the experimental setup in a similar manner in between sets of 40 experimental cages. The boxes were agitated via blowing and tapping like the experimental subjects, but the laser was not enabled. As during our previous anesthetized dosing, control mortality rates of 5% or less were deemed acceptable (i.e. if a given day’s controls showed greater than 5% mortality, those results were discarded).

After dosing or control treatments, the subjects remained in the boxes in which they were presented to the IFD apparatus and returned to the insectary within an hour. Subjects continued to have access to the same food sources as noted above. Mortality counts were performed at 24 + /−2 h from dosing or control exposure.

### Statistical considerations and analysis

For a given set of experiments, logistic regression was performed on mortality as a function of laser fluence using the generalized linear model framework within MATLAB. The quality of the model fit was assessed by the pseudo R^2^ value reported within this framework. The LD90 values were determined from this regression as well.

The number of subjects used for each laser condition within a given experiment was chosen as the best compromise between the sizes of resulting LD90 confidence intervals (using exact binomial distributions) and practical considerations on subject throughput. Using 40 subjects per data point offered throughput of 5–6 data points in a given week, typically sufficient to define a logistic regression curve with resulting confidence intervals for the LD90 parameter of ~ 10%. For some experiments, additional data points had to be acquired in subsequent weeks (with fresh sets of subjects) if the initial mortality outcomes did not provide data points sufficient to define a logistic curve.

For experiments that did not seek to establish dose–response curves, χ^2^ tests (contingency table approach) were used to compare whether mortalities were equivalent among the different test conditions.

For comparing mortality as a function of tracking error, or comparing tracking error as a function of pulse duration, Kruskal–Wallis tests were performed along with pair-wise follow-up testing. This non-parametric equivalent to ANOVA was selected since the data requirements for using ANOVA could not be verified for these datasets.

## Supplementary information

Supplementary Information 1.

Supplementary Video 1.

Supplementary Video 2.

Supplementary Video 3.

Supplementary Video 4.
