# Supplementary material for: Optical tracking and laser-induced mortality of insects during flight
Source: Sci Rep. 2020 Sep 9;10:14795. doi: 10.1038/s41598-020-71824-y (PMC7481216; doi:10.1038/s41598-020-71824-y)
Supplement: Supplementary file 1 — Supplementary Information 1. [file 41598_2020_71824_MOESM1_ESM.docx]

**Optical tracking and laser-induced mortality of insects during flight**

**Matthew D Keller*, Bryan J Norton, David J Farrar, Phil Rutschman, Maclen Marvit, Arty Makagon***

**Intellectual Ventures Laboratory, Bellevue, WA, USA**

***matt.keller.09@gmail.com**

***amakagon@PhotonicSentry.com (primary)**

**Supplemental Figures**


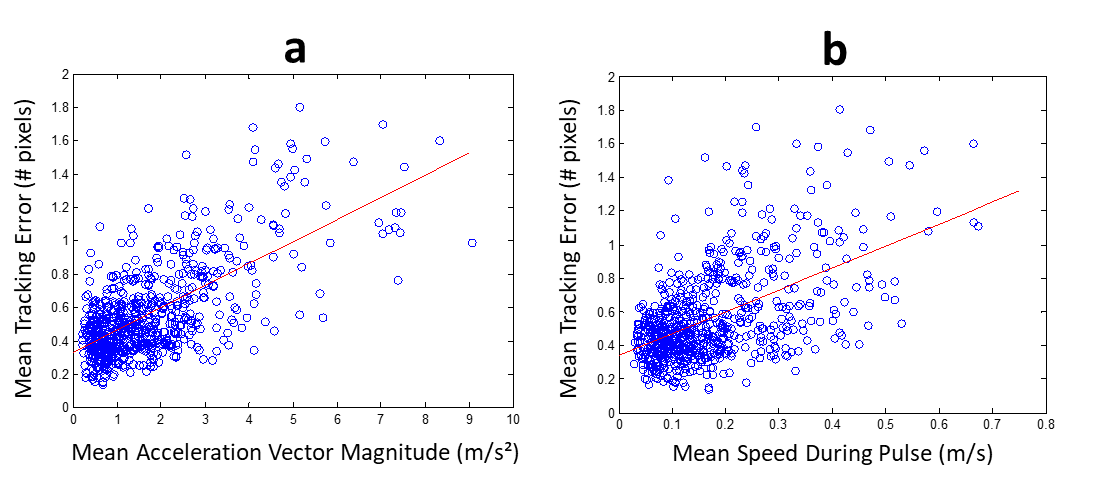


**Supplemental Figure S1.** Correlations between *A. stephensi* flight parameters and mean tracking error during all doses with 445 nm and 532 nm experiments shown in Table 2. Of the parameters shown in Table 1, only **(a)** acceleration vector magnitude (R^2^ = 0.44, p < 10^-91^) and **(b)** flight speed (R^2^ = 0.29, p < 10^-50^) showed significant correlations with mean and max tracking errors. Note that for all values of these flight parameters, the mean tracking error remained under our defined requirement of 2 pixels and max error remained under 3 pixels (not shown).


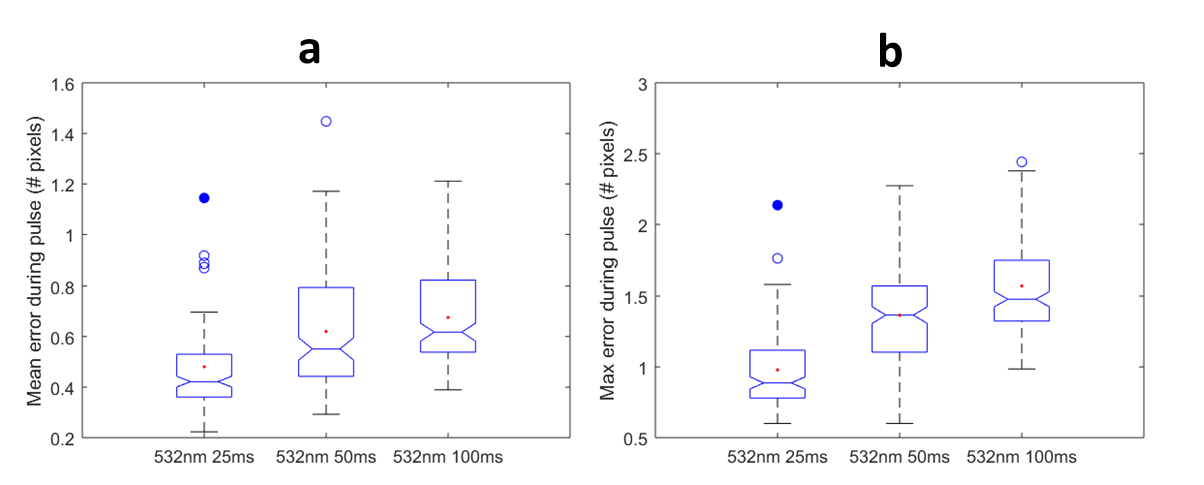


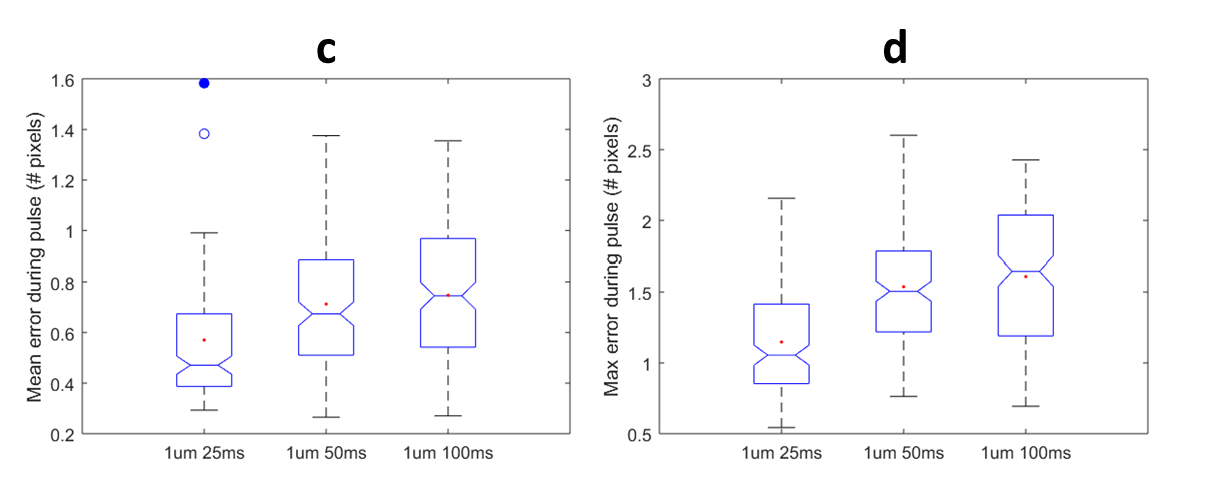


**Supplemental Figure S2.** Comparing tracking performance for the 25, 50, and 100 ms pulse durations from the constant pulse energy experiments. **(a)** The mean tracking errors for 532 nm experiments were not equivalent according to a Kruskal-Wallis test (p < 10^-5^); follow-up testing showed that the error for 25 ms data was distinct from the 50 and 100 ms data (p < 0.007 and < 10^-5^, respectively), while the latter two were statistically equivalent (p > 0.14). **(b)** Findings for the max tracking errors across pulse durations were similar, with p < 10^-10^ for comparing all three groups, the 25 ms error lower than for the other two pulse durations (p < 10^-5^), and p > 0.11 for comparing 50 and 100 ms errors. **(c, d)** Mean and max tracking errors during 1064 nm experiments followed the same pattern, with p < 0.001 and < 10^-4^ across all pulse durations, respectively. Follow-up testing showed the 25 ms results as distinct from 50 ms (p < 0.001 for mean and max) and from 100 ms (p < 0.001 for mean and < 10^-4^ for max). Comparing 50 and 100 ms tracking errors showed high equivalence for both mean and max (p > 0.74).

**Supplemental Video captions**

**Supplemental Video 1.** High speed video footage of initial IFD test using laser parameters of 532 nm, 2.5 mm spot size, and a 25 ms pulse with 3 W (the approximate LD90 for these conditions). Video captured at 2000 frames per second and played back at 1/67 speed. Laser dose corresponds to approximately the 2 to 3.5 sec time period, as noted by the “LASER ON” popup, as well as a faint red dot visible on and behind the target (likely due to autofluorescence from the subject since a notch filter blocked all 532 nm light from entering the high speed camera).

**Supplemental Video 2.** High speed video footage (captured at 2000 frames per second and played back at 1/67 speed) of IFD of ACP target. Laser conditions were 1064 nm, 2.5 mm spot size, and a 25 ms pulse with 25 W, which corresponded to the LD90 condition for mosquito subjects. The laser pulse is evident as the bright flash, which come from scattered 1064 nm light making it through the inherent Bayer filters in the camera.

**Supplemental Video 3.** High speed video footage (captured at 2000 frames per second and played back at 1/67 speed) of long-range IFD with the *A. stephensi* subject cage placed 30 m from the optical setup, as compared with the previous 2 m distance. Laser conditions were 1064 nm, 3.0 mm spot size, and a 25 ms pulse with 30 W, which provided nearly the same LD90 fluence compared with the shorter range setup. Laser dose is again visible as the white flash.

**Supplemental Video 4.** Identical 30 m IFD video footage to Video 3, but for a *C. pipiens* subject in place of *A. stephensi*. Note that this subject appeared to be dosed slightly closer to the edge of the cage than allowed for the shorter range system due to the relatively less-optimized setup for this system.
